# The magnitude and trend of artemether-lumefantrine stock-outs at public health facilities in Kenya

**DOI:** 10.1186/1475-2875-11-37

**Published:** 2012-02-08

**Authors:** Raymond K Sudoi, Sophie Githinji, Andrew Nyandigisi, Alex Muturi, Robert W Snow, Dejan Zurovac

**Affiliations:** 1 Malaria Public Health and Epidemiology Group, KEMRI/Wellcome Trust Research Programme, PO Box 43640, 00100 GPO Nairobi, Kenya; 2 Division of Malaria Control, Ministry of Public Health and Sanitation, P.O. Box 19982-00202, Nairobi, Kenya; 3 Management for Sciences of Health, P.O. Box 8700-00100, Nairobi, Kenya; 4 Centre for Tropical Medicine, Nuffield Department of Clinical Medicine, University of Oxford, CCVTM, Oxford, UK; 5 Center for Global Health and Development, Boston University, Boston, MA 02118, USA

## Abstract

**Background:**

Health facility stock-outs of artemether-lumefantrine (AL), the common first-line therapy for uncomplicated malaria across Africa, adversely affect effective malaria case-management. They have been previously reported on various scales in time and space, however the magnitude of the problem and trends over time are less clear. Here, 2010-2011 data are reported from public facilities in Kenya where alarming stock-outs were revealed in 2008.

**Methods:**

Data were collected between January 2010 and June 2011 as part of 18 monthly cross-sectional surveys undertaken at nationally representative samples of public health facilities. The primary monitoring indicator was total stock-out of all four weight-specific AL packs. The secondary indicators were stock-outs of at least one AL pack and individual stock-outs for each AL pack. Monthly proportions and summary means of the proportions over the monitoring period were measured for each indicator. Stock-out trends were assessed using linear regression.

**Results:**

The number of surveyed facilities across 18 time points ranged between 162 and 176 facilities. The stock-out means of the proportion of health facilities were 11.6% for total AL stock-out, 40.6% for stock-out of at least one AL pack, and between 20.5% and 27.4% for stock-outs of individual AL packs. Monthly decrease of the total AL stock-out was 0.005% (95% CI: -0.5 to +0.5; p = 0.983). Monthly decrease in the stock-out of at least one AL pack was 0.7% (95% CI: -1.5 to +0.3; p = 0.058) while stock-outs of individual AL packs decreased monthly between 0.2% for AL 24-pack and 0.7% for AL six-pack without statistical significance for any of the weight-specific packs.

**Conclusions:**

Despite lower levels of AL stock-outs compared to the reports in 2008, the stock-outs at Kenyan facilities during 2010-2011 are still substantial and of particular worry for the most detrimental:- simultaneous absence of any AL pack. Only minor decrease was observed in the stock-outs of individual AL packs. Recently launched interventions to eliminate AL stock-outs in Kenya are fully justified.

## Background

Universal and continuous availability of artemisinin-based combination therapy (ACT) is a critical pre-requisite for delivery of effective malaria case-management at health facilities across Africa [1]. In most African countries, information on the ACT availability at peripheral facilities is either absent or collected periodically during the cross-sectional surveys undertaken on various scales in time and space [2-5]. Yet, this limited information suggests that ACT stock-outs are common, however its trends over time are less clear. Furthermore, publicizing ACT stock-outs is a crucial element to raise awareness of this problem and initiate interventions aimed at elimination of ACT stock-outs at the point of care [6].

In Kenya, the nationally recommended ACT, artemether-lumefantrine (AL), was introduced to health facilities in 2006 as the first-line treatment for uncomplicated malaria [7]. In 2008, a cross-sectional survey undertaken at public facilities in seven Kenyan districts revealed that a total AL stock-out of all four weight-specific packs was present at 26% of facilities while 75% of facilities were stocked out of at least one AL pack [8]. In 2009, the new National Malaria Strategy and Monitoring and Evaluation Plan 2009-2017 was launched and specified that by 2013, all facilities should have AL continuously in stock [9]. While the 2008 findings were alarming, it has also been recognized that facility-based surveys undertaken periodically on limited geographical scale in nationally non-representative districts are no longer adequate to timely monitor national trends in the AL availability. Moreover, although integrated within essential medicines supply, Kenyan AL supply chain for government facilities is further complicated by quarterly distribution of AL based on consumption to rural health facilities in three out of eight provinces and every two months to all hospitals countrywide ("pull-system"), while rural facilities in the remaining five provinces receive predetermined quantities of AL every three months ("push-system"). In contrast to government facilities, "pull-system" is a predominant, but not an exclusive, distribution system for faith-based facilities. Therefore, from 2010 onwards, monthly monitoring of AL availability on the nationally representative sample of facilities was initiated. In this brief report, the national findings in the period between January 2010 and June 2011 are presented and the new interventions aiming to eliminate AL stock-outs in Kenya by 2013 are highlighted.

## Methods

### Indicators

The indicators reflecting AL stock-out included the stock-out of each of four weight-specific AL packs (six, 12, 18 and 24 tablets), stock-out of at least one of the four AL packs and the total stock-out of all four AL packs. Since the total AL stock-out is the most detrimental because it precludes any AL treatment at the health facility, the primary monitoring indicator was total AL stock-out defined as simultaneous absence of all four weight-specific packs on the survey day.

### Sampling

The detailed explanation of the facility sampling methods is presented elsewhere [10,11]. Briefly, from the universe of public health facilities in Kenya, a national representativeness was assured, drawing a random sample stratified by administrative boundaries, type of facilities and their ownership. In each of seven surveyed provinces, four strata based on the facility type and ownership was formed. Subsequently, from each of the 28 strata, a simple, random sample proportional to the number of facilities in a stratum was drawn. The estimated sample size of 170 facilities was sufficient to obtain 95% confidence intervals of ± 7.5% around a conservatively estimated national stock-out frequency of 50%.

### Data collection and analysis

Data were collected on a monthly basis between January 2010 and June 2011 using cross-sectional surveys undertaken at the nationally representative sample of public health facilities. Two rounds of physical surveys were undertaken to assess availability of commodities and malaria case-management practices, after which AL stock-out data were collected through follow-up phone call interviews with in-charges of the same facilities. Case-management findings from physical surveys are reported previously [11] while here AL stock-out data combining physical and phone call surveys are reported. The analysis was descriptive, reporting national level estimates and included stand-alone monthly proportions and summary means of the proportions for each indicator across an 18-month monitoring period. Stock-out trends over time were assessed for each indicator using linear regression analysis. Analysis was undertaken in Excel and STATA 11. Ethical approval was provided by the Kenyatta National Hospital/University of Nairobi-Ethics and Research Committee (KNH-ERC/A/383). Informed written consent was obtained for all participants.

## Results

**Figure 1:**
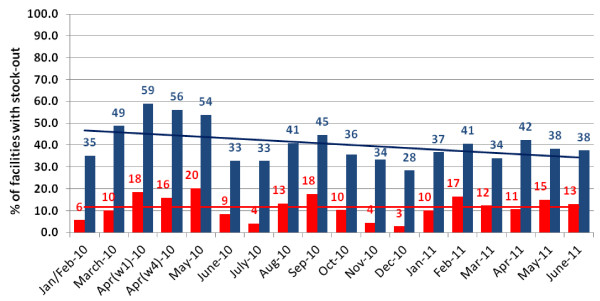
**AL stock-out at Kenyan facilities between January 2010 and June 2011 (red bars showing total AL stock-out, blue bars showing stock-out of at least one AL pack, and red and blue lines showing trends over time for respective stock-out indicators)**.

The number of surveyed facilities across 18 time points ranged between 162 and 176 facilities. Figure 1 shows monthly proportions and trends in stock-outs of at least one AL pack and simultaneous stock-out of all four AL packs (total AL stock-out). Mean of the proportion of health facilities with total AL stock-out across the study period was 11.6% (median 11.5%; monthly range: 3-20%) while mean stock-out of at least one AL pack was substantially higher -40.6% (median 38.0%; monthly range: 28-59%). AL stock-outs had shown a fluctuating pattern however overall changes in AL stock-outs during the monitoring period were minor. Over an 18-month period, monthly decrease of total AL stock was 0.005% (95% CI: -0.5 to +0.5; p = 0.983) while monthly decrease in the stock-out of at least one AL pack was 0.7% (95% CI: -1.5 to +0.3; p = 0.058).

With respect to the stock-outs of individual AL packs over the study period, mean of the proportion of stocked-out facilities ranged between 20.5% for AL six-pack (median 19.4%; monthly range: 9-39%) and 27.4% for AL 18-pack (median 26.6%; monthly range: 14-43%). The mean stock-out of AL 12- and AL 24-packs was respectively 27.2% (median 26.4%; monthly range: 14-48%) and 22.4% (median 21.6%; monthly range 13-32%). There was a minor, however statistically insignificant, declining stock-out trend for all weight specific AL packs during the monitoring period. Monthly decreases were 0.7% for AL six-pack (95% CI: -1.6 to +0.1; p = 0.082), 0.5% for AL 12-pack (95% CI: -1.5% to +0.5%; p = 0.326), 0.4% for AL 18-pack (95% CI: -1.1 to +0.3; p = 0.266) and 0.2% for AL 24-pack (95% CI: -0.3 to +0.7; p = 0.442).

## Discussion

ACT stock-out trends from a nationally representative sample of public health facilities are reported for the first time in Africa. The study reveals important findings quantifying the magnitude of the problem in Kenya, emphasizing the importance of interventions to eliminate stock-outs in the country. The results of 18 months' monitoring period in 2010-2011 revealed that on average 12% of facilities had total AL stock-out, 41% were missing at least one AL pack, and stock-outs of individual weight-specific packs ranged from 21 to 27%. On the positive side, these stock-out levels are significantly lower than reported in 2008 when one quarter of facilities was found without any AL and as high as three quarters were without one or more AL packs [8]. The stock-out indicators in each month during the 18-month monitoring period were lower than observed in 2008 in Kenya and indeed lower than reported in several larger and smaller cross-sectional surveys in other African countries [2-5]. The lower levels of AL stock-outs in the recent period are likely to be due to higher stocks at the central level and less common procurement delays that massively compromised AL delivery and distribution in 2008 [8,12].

Nevertheless, the levels of current AL stock-outs are still substantial and trends do not show significant improvements. Some fluctuations observed between months are likely a reflection of the drug distribution cycles with more facilities running out of stock towards the end of the cycle. The stock-out levels are of particular concern for the total absence of any AL pack and the finding that over one in 10 health facilities countrywide are unable to deliver life-saving therapy for malaria patients is worrying. As recently observed in the Western Kenya, the total AL stock-out has been indeed associated with increased childhood mortality [13]. Beside the total stock-out, the stock-outs of individual AL packs also deserve attention. Although the strength of AL tablets is the same, four different pack sizes - each accompanied with pictorial instructions on AL use - are adequate for the management of four different weight categories of patients. Improvising AL treatments by cutting larger pack sizes for lower weight categories or combining smaller packs for heavier patients still enables AL dispensing however it may compromise previously observed high levels of patients' adherence [14,15] and treatment outcomes [16]. Indeed several recent studies undertaken under the routine conditions of care reported high levels of non adherence [17-20] and the possible negative effects of the stock-outs of the weight-specific packs on patients' adherence merit further investigations.

The investigations of the causes of AL stock-outs are beyond the scope of this monitoring exercise and qualitative and quantitative studies investigating the complete supply chain for medicines are necessary to comprehensively understand these problems. Yet, acknowledging the persistence of AL stock-outs in the country, the Kenyan Division of Malaria Control has reinforced programmatic activities to strengthen the supply chain for anti-malarial drugs of which those targeting peripheral health workers and district managers are of particular interest. Beside the on-going activities aiming at improvements of the routine logistic information systems through the in-service training of health workers, supportive supervision and promoting peripheral redistribution of commodities, in August 2011 a real-time reporting of AL availability using mobile phone text-messaging was launched in five pilot districts. The project is following Tanzanian "SMS for Life" model which had shown a drastic reduction of AL stock-outs by ensuring real-time visibility of stocks that in turn resulted in interventions mitigating stock-outs [21]. In Kenya however, it has been recognized that causes of AL stock-outs are not only a result of suboptimal supply and peripheral drug management but also a consequence of irrational use of anti-malarial medicines due to low testing rates and suboptimal interpretation of test results in clinical practice [10,11]. Therefore, to optimize potential success of the intervention, the Kenyan "SMS for Life" project decided to mimic Tanzanian model with the respect to weekly reports of four AL packs but also, alongside the scaling up process of malaria diagnostics, to include stock reports of rapid diagnostic tests and basic surveillance information on testing and treatment parameters. It is hoped that in the era of imperfect drug supply the package of interventions, including intense monitoring with high visibility at all levels of care and adequate local responses, may be sufficient to enable Kenya to eliminate stock-outs and achieve 2013 targets of universal and continuous AL availability. Furthermore, if this impact could be obtained through real-time reporting on the national scale there will be obviously a reduced need for repeated national surveys to monitor trends. Finally, it should be acknowledged that interventions at district levels such as these initiated in Kenya, have potential only if major stock-outs at the central level do not occur. Fortunately, this potential, higher level problem of the supply chain, despite some deficiencies that are beyond the scope of this report, has not been encountered recently on a major scale in Kenya.

## Conclusions

Lower levels of AL stock-outs during 18 months of monitoring period in 2010-2011 were found than previously reported in Kenya. However, 2010-2011 stock-out levels are still substantial and without significant improvements over time. In Kenya, as well as in other African countries, monitoring and reporting of ACT stock-outs should be a regular activity, important to inform national policies and donors on the progress in the implementation of anti-malarial drug policies but also to raise awareness and initiate interventions to potentially mitigate this problem.

## Competing interests

The authors declare that they have no competing interests.

## Authors' contributions

All authors contributed to the study design, data analysis, interpretation of the results, and drafting and finalization of the manuscript. All authors read and approved the final manuscript.
